# Mechanisms of Metal Resistance and Homeostasis in Haloarchaea

**DOI:** 10.1155/2013/732864

**Published:** 2013-02-21

**Authors:** Pallavee Srivastava, Meenal Kowshik

**Affiliations:** Department of Biological Sciences, Birla Institute of Technology and Science, Pilani, K K Birla Goa Campus, NH-17B, Zuarinagar, Goa 403 726, India

## Abstract

Haloarchaea are the predominant microflora of hypersaline econiches such as solar salterns, soda lakes, and estuaries where the salinity ranges from 35 to 400 ppt. Econiches like estuaries and solar crystallizer ponds may contain high concentrations of metals since they serve as ecological sinks for metal pollution and also as effective traps for river borne metals. The availability of metals in these econiches is determined by the type of metal complexes formed and the solubility of the metal species at such high salinity. Haloarchaea have developed specialized mechanisms for the uptake of metals required for various key physiological processes and are not readily available at high salinity, beside evolving resistance mechanisms for metals with high solubility. The present paper seeks to give an overview of the main molecular mechanisms involved in metal tolerance in haloarchaea and focuses on factors such as salinity and metal speciation that affect the bioavailability of metals to haloarchaea. Global transcriptomic analysis during metal stress in these organisms will help in determining the various factors differentially regulated and essential for metal physiology.

## 1. Introduction

Many metal ions have a key role in the physiology of cells. Metals such as calcium, cobalt, chromium, copper, iron, potassium, magnesium, manganese, sodium, nickel, and zinc are essential and serve as micronutrients. These metals act as the redox centers for metalloproteins such as cytochromes, blue copper proteins, and iron-sulfur proteins which play a vital role in electron transport [1]. As the transition metals exist in numerous oxidation states, they efficiently act as electron carriers during redox reactions of electron transport chains to generate chemical energy [2, 3]. Metal ions also function as cofactors and confer catalytic potential and stability to proteins [4]. Other metals like silver, mercury, lead, aluminum, cadmium, gold, and arsenic have no biological roles and are potentially toxic to microbes [5]. The toxicity is exerted by the displacement of essential metals from native binding sites or through ligand interactions [6]. Both essential and nonessential metals at high concentrations disrupt cell membrane, alter enzymatic specificity, hinder cellular functions, and damage DNA [5]. Thus, as any disturbance in metal ion homeostasis could produce toxic effects on cell viability, the concentrations of metals within cells are stringently controlled. An increase in the ambient metal concentration leads to activation of metal resistance mechanisms to overcome metal stress. Metal homeostasis has been well studied in bacteria and eukaryotes and is attributed to differential regulation of transporters like P_1B_-type ATPases, ABC transporters, cation diffusion facilitators (CDFs), and metallochaperones in response to metals [7–9]. Among the Archaea, thermophiles and hyperthermophiles of the Crenarchaeota and the methanogens and thermophiles of Euryarchaeota utilize P_1B_-type ATPases and ABC transporters for metal transport and homeostasis [10, 11]. However, metal homeostasis in haloarchaea from the phylum Euryarchaeota has not been extensively studied [11]. 

Haloarchaea are members of the third domain of life, the Archaea, within which a total of 36 genera and 129 species have been identified to date [12]. These organisms require between 10% and 35% salt for optimum growth and are the predominant microflora of hypersaline environments such as solar salterns, salt lakes, soda lakes, salt deposits, and so forth [13]. However, some low salt tolerant haloarchaea can be found in estuarine environments [14]. Estuaries serve as interfacial mixing zones between rivers and seawaters which determine the flux of chemical species into the ocean [15]. Econiches like estuaries [16] and solar crystallizer ponds [17] may contain high concentrations of metals, since they serve as ecological sinks for metals and as effective traps for river borne metals [18]. Anthropogenic activities like urbanization and industrialization, including mining, agriculture, and waste disposal, further contribute towards metal pollution at these sites [19, 20]. Haloarchaea have developed various mechanisms of resistance in order to thrive under metal stress [21–23]. However, studies on metal resistance in haloarchaea are still in their infancy.

Most of the reports until now are limited to MIC (minimum inhibitory concentration) studies [24–26]. However, in a study on comparative gene analysis of the role of P_1B_-type ATPases in maintaining metal homeostasis in bacteria and archaea, Coombs and Barkay (2005) [10] have shown that P_1B_-type ATPases containing N-terminal metal-binding motifs are distributed across bacteria and archaea, including haloarchaea. These ATPases along with the ABC transporters, transcriptional regulators, and certain metallochaperones were found to be involved in metal resistance and homeostasis in the haloarchaeon *Halobacterium *sp. strain *NRC-1 * [22]. Haloarchaea exhibit a high degree of variation in the concentration of metals that they can tolerate [22, 24–26]. Interestingly, at low concentrations, certain metal ions like Mn(II), Fe(II), Co(II), Ni(II), and Zn(II) were found to enhance growth [22, 24]. An in-depth study at molecular level may help in better understanding of this variation. This paper seeks to give an overview of the main molecular mechanisms involved in metal tolerance in haloarchaea and to outline the factors such as salinity and metal speciation that affect the bio-availability of the metals to haloarchaea. The need for further studies on metal homeostasis and resistance in haloarchaea is highlighted. The elucidation of complete pathways of metal resistance from uptake to transformation/detoxification and efflux will help to determine the final fate of metals. The final metal species could be either volatile or chelated intracellularly and thereby rendered nontoxic to the organism. 

## 2. Bioavailability of Metals to Haloarchaea

For a metal to act either as a micronutrient or as a toxicant, it has to be available for uptake by the organism [27]. The metal species determines the solubility, bio-availability, and membrane transport, besides influencing the phenomenon of adsorption, oxidation/reduction, and oceanic residence times [28]. Metal speciation is governed by alkalinity, pH, hardness (presence of Ca/Mg), natural dissolved organic matter, redox potential, and salinity [29]. Strongly complexed and thus nonlabile and particulate metal species are less available to organisms for uptake [30]. As haloarchaea inhabit hypersaline environments with salinity in the range of 2%–35%, salinity is proposed to be the most important factor affecting bio-availability. The salt content in hypersaline econiches of solar salterns is about 10-fold of its concentration in seawater [31] due to evaporation. This process also concentrates other anions and cations present in the sea water including the metal salts fed through contaminated estuaries [32]. The high chloride ion (Cl^−^) content in these environments results in the formation of metal chlorocomplexes. 

The type of complex formed depends upon the chelating ligand, that is, organic or inorganic ligands, and the kind of heavy metal present in the system [33]. Metals like Zn and Cu that have small ionic radii preferentially complex with hard donors containing oxygen like OH^−^, CO_2_^3−^, HCO_3_^−^, and SO_4_^2−^ to form inorganic complexes [34]. Soft acceptors like Hg, Cd, and Ag are easily ionized and are thus, more likely to form chlorocomplexes. Although inorganic species exist in natural waters, organic metal species predominate [35]. The complexation of metals with organic ligands reduces bioavailability as organic-metal complexes are not readily transported across cell membranes [30, 36]. Inorganic species, on the other hand, are readily available to the biota as the complexes are weak and dissociate rapidly to form free ions which bind to the transporters or are chelated by biotic ligands secreted by the organisms [37–40].  Table 1 shows the type of inorganic species formed at different salinities for five major metals. 

While metal bio-availability, uptake, and toxicity decrease in presence of natural dissolved organic ligands, metals differ in their behavior at high salinities. For example, in case of cadmium, speciation is highly dependent on the complexing ligands. In river water it exists either as CO_3_^−^ complex or free cation, and in oceanic waters it exists as highly soluble CdCl_2_, whereas in estuarine waters, it forms a strong CdCl^+^ complex which is biologically unavailable [41–43]. In case of silver, insoluble AgCl^0^ is formed in estuarine and oceanic waters, while under hypersaline conditions, soluble AgCl^2-^, AgCl_3_^2−^, and AgCl_4_^3−^ complexes are formed [44]. Soluble HgCl^−^ and sparingly soluble HgCl_2_ are the predominant complexes of Hg at high salinities [45]. HgCl_2_ and the soluble silver-chloro complexes are lipophilic and can easily diffuse through cellular membranes [46]. Zn and Cu exist as ZnCl^+^ and CuCl^+^ which coprecipitate at higher salinities, due to decrease in the net negative charge on macromolecular suspended particles and therefore are not available for uptake. Unlike Zn(II) and Cu(II), Fe(II), Co(II), Ni(II), and Mn(II) form weak complexes with Cl^−^, that easily dissociate and can be taken up by organisms [28].  Table 2 summarizes the bioavailability of metal-chloro complexes. 

The bioavailability of chlorocomplexes also depends upon the type of biotic ligands present. Biotic ligands are the receptors on an organism where metal binding takes place which results in the manifestation of its toxic effects [47]. Metal receptors and ion transporters such as Na(II) and Ca(II) transporters present on fish gill surfaces, algal membranes, phytoplankton membranes, and so forth act as biotic ligands [47]. Binding of metals to biotic ligands is unaffected by changes in salinity. However, metal complexes adsorbed to abiotic ligands such as sediments are desorbed with increase in salinity. Thus, biotic ligands render the metals unavailable to other organisms for uptake. For example, in case of silver, with increase in salinity, desorption of Ag(I) complexed with suspended sediments and formation of soluble chlorocomplexes, which are bioavailable have been observed. However, biosorbed Ag(I) is not influenced by the increase in salinity, and desorption of Ag(I) does not occur [48].

The toxicity of a metal to microorganisms does not have a linear relationship with its concentration, and it depends strongly upon chemical speciation [49, 50]. For certain metals such as Zn(II) and Cu(II), complexation with chloride ions may result in precipitation at high salinities. Therefore, these complexes are not available to micro-organisms for uptake. However, metals such as Hg(II), Ag(II), Fe(II), Co(II), Ni(II), and Mn(II) either form lipophilic soluble chlorocomplexes or weak chlorocomplexes that dissociate easily and are thus available to organisms for uptake. Therefore, while studying metal resistance in haloarchaea, metal speciation and the bio-availability of metals should be taken into consideration.

## 3. Metal Resistance

**Figure 1:**
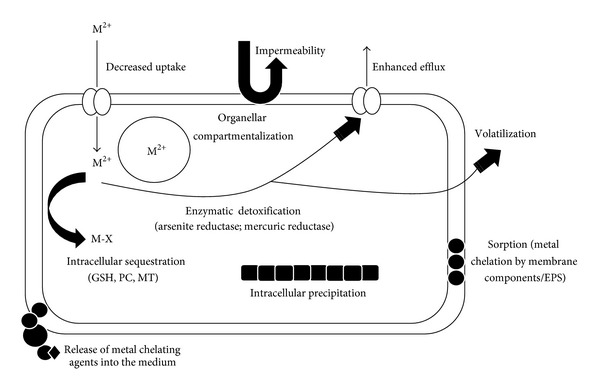
General mechanisms adapted by bacteria, eukaryotes, and archaea for metal resistance. All the three domains exhibit sorption of metals, volatilization, release of metal chelating compounds in the medium, enhanced efflux, impermeability, decreased uptake, enzymatic detoxification, and intracellular chelation as mechanisms for metal resistance. Organellar compartmentalization is observed only in eukaryotes, with the exception of magnetosomes in magnetotactic bacteria.

Organisms inhabiting the metal polluted environments develop resistance mechanisms that enable efficient detoxification and transformation of toxic forms to nontoxic forms. The majority of bacteria and eukarya tolerate metals by a reduced influx/enhanced efflux [51, 52] or enzymatic detoxification sometimes followed by volatilization [6, 53]. Intracellular compartmentalization is observed only in eukaryotes [51].  Figure 1 shows the various mechanisms of metal resistance exhibited by all the three domains of life. 

Intracellular chelation (Figure 1) by a variety of cysteine- (Cys-) rich metal-binding peptides like glutathione (GSH) and proteins like metallothioneins (MTs) and phytochelatins (PCs) also confers resistance to metals in many microbes [54]. MTs are genetically coded small molecular weight polypeptides that are classified based upon the number of Cys residues [55]. They typically have two Cys-rich domains that bind heavy metals through mercaptide bonds, giving these proteins a dumbbell-shaped conformation comprising an N-terminal *β*-domain that usually binds 3 metal ions and a C-terminal *α*-domain that binds 4 metal ions [56, 57]. PCs comprise (*γ*-GluCys)_n_-Gly where *n* is usually in the range of 2 to 5. They are enzymatically synthesized by PC synthase using GSH as the substrate [58, 59]. The thiol group of the cysteine residue in PCs sequesters heavy metals. Apart from these cysteine-rich peptides, cells may secrete other metal sequestering proteins like siderophores and DNA-binding protein from nutrient starved cells (Dps) (Figure 1). Siderophores are a class of low molecular weight iron chelating compounds which store iron and are overexpressed during conditions of stress or iron deficiency [60]. They are chemically diverse and generally possess oxygen-donor-type chelating functional groups [61]. Once chelated, the Fe-siderophore complex is transported to the periplasm through the energy-coupled transport involving TonB dependent transporters (TBDT) and the inner membrane TonB-complex, composed of TonB, ExbB, and ExbD. The Fe-siderophore can then be transported to the cytoplasm through ABC transporters like ferrichrome or permeases [62]. TonB protein is responsible for transducing cytoplasmic membrane energy to the outer membrane which results in TonB associating with or changing its affinity towards the outer membrane, while ExbB/D components antagonize this association or affinity of TonB to cytoplasmic membrane [63].Siderophores have also been shown to chelate metals other than Fe [62]. Dps are structurally homologous to ferritins, the primary iron storage/detoxification proteins, are usually expressed in response to excess of iron, and are found in all three domains of life [64]. These proteins have pores that are lined with acidic residues that bind cations like Fe(II) [65]. The binding of Fe(II) to Dps protects cells from oxidative stress by inhibiting the Fe-catalyzed production of hydroxyl radicals [66]. 

Haloarchaea have *γ*-glutamylcysteine (*γ*-GC) [67, 68] which is analogous to GSH and is involved in maintaining a reducing environment within the cell, overcoming oxidative and disulfide stress and detoxification of xenobiotics [69]. The thiol group of cysteine in *γ*-GC can chelate the toxic metal ions thereby conferring resistance. A unique phenomenon observed in archaea is the heavy metal-induced multimerization of metal chelating proteins such as CutA- and DpsA-like proteins that result in the precipitation of the protein-metal ion complex [23]. This precipitate resolubilizes and the multimers disintegrate when the metal stress decreases [70, 71]. Although these proteins are known to be involved in divalent metal tolerance in bacteria and eukaryotes, the multimerization of these proteins has been observed only in archaea. The aspartate residue in position 48 has been found to be critical for metal-induced multimerization and metal ion binding of CutA protein in *Pyrococcus horikoshii. *Substitution of Asp48 with alanine decreases the amount of aggregate formation [70]. Similarly, the multimeric non-haem ferritin DpsA-like protein of *Halobacterium salinarum* ensued from an assembly of 12 units and was found to sequester iron in response to the oxidative stress exerted by excess iron [72]. This protein was downregulated under iron-deficient conditions unlike the other *dps* that are upregulated under these conditions. It exhibits the features of non-haem bacterial ferritins that are expressed to sequester the excess iron. Their expression is repressed under conditions of iron starvation [73]. Overexpression of siderophores in haloarchaea may increase chelation in case of iron deficiency. On the other hand, repression of these siderophores in presence of excess iron may avoid uptake [68, 74]. MTs are absent in archaea [23]. 

Biosorption of metals by the organisms at the surface or by the exopolysaccharides (EPS) secreted to form the biofilms enables organisms to tolerate metals [75, 76]. Biofilm forming organisms exhibit an altered phenotype with respect to growth rate and gene transcription [77]. Haloarchaea synthesize EPS as a protective mechanism for survival under adverse conditions such as nutrient starvation, temperature fluctuation, and presence of toxic compounds [78]. Similarly the hyperthermophilic archaeon, *Archaeoglobus fulgidus, *was found to form a biofilm in response to toxic concentrations of metals, where the toxic metal was proposed to be trapped within the EPS matrix [78]. Thus, it is probable that under metal stress, haloarchaea may secrete EPS to make the cell impermeable to metals. In a study by Kawakami et al. (2007) [79], it has been found that *Halobacterium salinarum *CCM 2090 has a Ca(II)-dependent aggregation system, where the Ca(II) binds to certain aggregation factors present on the cell surface and induces ionic crossbridging between the EPS resulting in aggregation of the haloarchaeal cells. The presence of certain receptor proteins on the cell surface that interact with Ca(II) to form cell aggregates/flocs has also been demonstrated [79]. Four haloarchaeal genomes, *Haloquadratum walsbyi, Haloarcula marismortui, Haloterrigena turkmenica,* and *Halobacterium *sp. strain* NRC-1, *have been annotated with *cbp* encoding the cell surface calcium-binding acidic-repeat protein [80–83] that has been proposed to be the factor involved in Ca(II)-dependent aggregation, although its role in this process remains to be demonstrated. A similar dependence on Ca(II) and/or Fe(II) for biofilm formation is observed in *Vibrio cholerae *[84] and *Pseudomonas aeruginosa *[85]. Ca(II) is the twentieth element found in the fourth row of the periodic table, which could be replaced by other transition metal ions such as Mn(II), Cr(II), Fe(II), Co(II), Ni(II), Cu(II), and Zn(II), belonging to the same row. The distinctive electronic configuration of these metals, characterized by preferential filling of the 4s subshell before the 3d subshell, may be responsible for these metals substituting Ca(II) during aggregate formation [79]. Thus, tolerance to these metals may be mediated through binding with EPS. This view is supported by the observation that there is no aggregation in presence of certain other metals lacking this electronic configuration, such as Mg(II) and Sr(II) (alkali earth metals), Mo(II), Cd(II) and Sn(II) (fifth period), and Hg(II) and Pb(II) (sixth period) [79]. Aggregation in haloarchaeal cells results in formation of nonadherent floating multicellular aggregates which is different from biofilm formation, where the adherent multicellular structures are attached to diverse surfaces [86]. Recently, biofilm formation involving EPS (glycoconjugates and extracellular DNA) matrix was demonstrated in five haloarchaeal genera, *Halobacterium, Haloferax,* haloalkaliphilic* Halorubrum, *psychrotolerant *Halorubrum, *and a new genus of psychrotolerant haloarchaea isolated from Deep Lake, Antarctica [87]. Here Ca(II) ion did not have an effect on surface adhesion, suggesting the involvement of flagellar-twitching-motility-induced cellular aggregation and adhesion to substratum. Biofilm formation by other archaea like *Pyrococcus furiosus, Sulfolobus solfataricus,* and *Methanococcus maripaludis* involves cellular appendages such as archaeal type IV pili [88–90]. These are similar in structure and function to the type IV pili present in bacteria that facilitate cell-cell interactions, surface adhesion, and motility [91]. Archaeal type IV pili have been shown to be involved in biofilm formation by *Haloferax, Halobacterium, *and *Halorubrum* [87, 92, 93]. The biofilm formed may trap the metals within the EPS matrix and prevent the diffusion of metals inside the cell, thus conferring resistance to haloarchaea. 

Most bacteria carry the resistance determinants for metals as operons, on their plasmids [94]. The metal resistance operons usually include genes for transporters and an enzyme for detoxification. Haloarchaea exhibit resistance mechanisms similar to those of bacteria. The *ars* operon conferring arsenite and arsenate resistance in *Halobacterium *sp. strain* NRC-1* is present on one of its two plasmids [83]. A comparative genome analysis of bacteria and archaea revealed some common elements responsible for maintenance of metal homeostasis and resistance. P_1B_-type ATPases involved in cation transport with a high diversity in the N-terminal metal-binding motifs were found to be distributed throughout the bacterial and archaeal lineages [10]. Genome of *Halobacterium salinarum* NRC-1 was found to carry two distinct phylogenetic clusters, CopA1 (Cu(II) influx) and CopA2 (Cu(II) influx and efflux). These clusters were also found to span the entire diversity of the bacterial domain. Coombs and Barkay (2005) [10] have proposed that variation in N-terminal metal-binding motifs does not affect the metal translocation function of P_1B_-type ATPases and therefore concluded that divergence in consensus sequence of the N-terminal metal-binding motif might have been tolerated during evolution [10, 83]. But this is just one of the few studies on metal homeostasis in archaea. Similar studies understanding the phylogenetic variation within the family Halobacteriaceae will enable a better understanding of metal homeostasis, by giving a snapshot of substrate specificity, variation in active sites, and so forth [23].

### 3.1. Operons in Metal Resistance

Many metal resistance determinants have been characterized in the bacterial system [95–105], of which *mer* operon for mercury resistance [95], *ars* for arsenic resistance [97, 98], and *cad* operon for cadmium resistance [99] have been extensively studied. All archaea except haloarchaea have been shown to carry such metal resistance determinants [106, 107]. The most comprehensively studied among these are the *mer *operon of the thermoacidophilic archaeon *Sulfolobus solfataricus *[108, 109], *ars *operon of acidophilic archaeon *Ferroplasma acidarmanus *Fer1 [110] and *Thermoplasma acidophilum *[111], and the *cop* operon for copper resistance of *Sulfolobus solfataricus *P2 [112–114]. Although many heavy metal transporters like CbiNOQ, HemUV, NosFY, and so forth are present in haloarchaea, their arrangement in an operon has not been shown, except for the ArsA ATPase transporter as a part of *ars *operon for arsenic resistance in *Halobacterium* sp. strain *NRC-1* [21].

Most haloarchaea have large plasmids in addition to their genomes (chromosomes) known as minichromosomes/megaplasmids. These minichromosomes harbor genes for antibiotic resistance or metal resistance that may be essential for haloarchaeal survival [115]. The pNRC100, one of the two minichromosomes of model organism *Halobacterium *sp. strain *NRC-1,* harbors the *arsADRC* gene cluster, responsible for conferring arsenate (As(V)) and arsenite (As(III))/antimonite (Sb(III)) resistance [83]. A fifth gene for arsenic resistance, *arsB, *is present on the main chromosome. The *arsADRC* operon was annotated for As(III) transport due to its homology to previously characterized genes [116], but later, by gene knockout studies, it was shown to confer resistance to As(III) and Sb(III) [21]. As(V) can be taken up by the cells through phosphate transporters (pit/pst) and As(III) by aquaglycerophorins (glycerophorin membrane transport proteins) [117] or hexose transporters [118]. As(V) is then converted to As(III) by arsenate reductase encoded by *arsC* [119]. *arsA* codes for *P*_1B_-type ATPase transporters that help in extrusion of As(III)/Sb(III) from the cell. *arsR *and* arsD* encode trans-acting repressors of the operon. ArsR and ArsD bind to As(III)/Sb(III) resulting in expression of the *arsA *and *arsC*. Arsenate reductase encoded by *arsC* is expressed weakly in *Halobacterium *sp. strain *NRC-1,* and therefore deletion of *arsC *and *arsADRC *was found to be ineffective in conferring arsenate sensitivity [21]. The operon *arsADRC* was found to be inducible by arsenite and antimonite [21].

**Figure 2:**
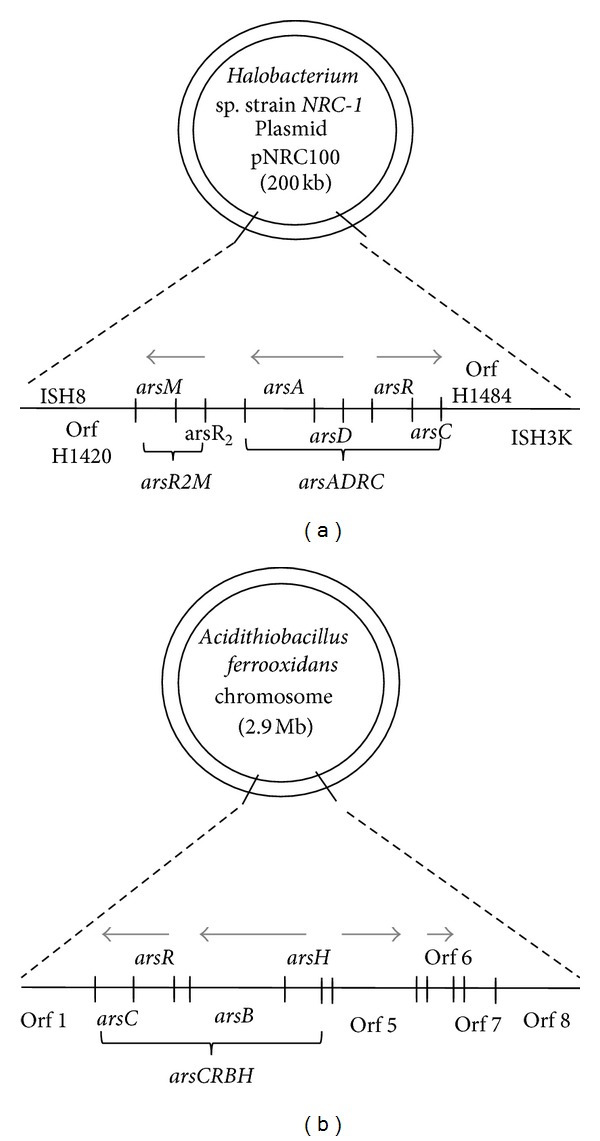
Arsenic resistance is determined by the presence of *ars* operon, which codes for an arsenite P_1_-type ATPases transporter ArsA/ArsB, an arsenate reductase ArsC, and arsenite responsive repressors ArsD and ArsR. The *arsADRC *and *arsR2M* operons are present on the plasmid in haloarchaeon *Halobacterium *sp. strain* NRC-1 *(a). The acidophilic bacterium *Acidithiobacillus ferrooxidans* has chromosomally encoded *arsCRBH *(b). The unique feature of these operons is the bidirectional nature transcription.

In bacteria, ArsB, an inner membrane protein, along with ArsA, the membrane-bound anion-transporting ATPase, forms the anion-conducting channel for arsenite extrusion [120]. Halobacterium sp. strain NRC-1 also harbors both arsA in ars operon on the megaplasmid pNRC100 and arsB on the main chromosome. However, arsB was found to play no role in arsenic resistance in this organism. Thus, it has been proposed that *Halobacterium* sp. strain *NRC*-1 harbors a novel transporter unrelated to ArsB but with a similar function [21]. Arsenic resistance in the Gram-negative acidophilic bacterium Acidthiobacillus ferrooxidans is determined by the chromosomally located arsCRBH operon comprising four genes [121]. The unique and common feature between the arsADRC and arsCRBH operons is the bidirectional nature of translation; that is, the arsAD and arsCR genes are translated in an opposite direction to arsRC and arsBH, respectively [21, 121] (Figure 2).

In *Halobacterium *sp. strain *NRC-1, *a second arsenite resistance operon, *arsR2M,* is present upstream of *arsADRC* on pNRC100 (Figure 2), where *arsR2 *is constitutively expressed while As(III)/Sb(III) induce the expression of *arsM *[21]. The *arsR2 *is analogous to *arsR* and *arsM* encodes a putative As(III)-methyltransferase very similar to human methyltransferases and S-adenosyl methionine-dependent methyltransferases of *Magnetospirillum magnetotacticum. *ArsM is involved in converting As(III) to methylated species like dimethylarsinate (DMA), trimethylarsine oxide (TMAO), or trimethylarsine (TMA) gas [122]. Deletion of *arsM* exhibited as increased sensitivity to arsenite but not towards arsenate or antimonite [21]. Thus, two possible mechanisms of As(III) resistance have been proposed to be conferred by *arsM*. First, the generation of a concentration gradient results in the movement of methylated arsenite (negatively charged/uncharged) out of the cell. Second, the volatile trimethylarsine formed diffuses out of the cell thus eliminating As(III) [123, 124]. Although arsenite methylation as a resistance mechanism is present in bacteria, *arsM* gene functions independently and is not a part of *ars* operon [123]. However, in *Halobacterium *sp. strain *NRC-1, *the *arsM *gene is present as a part of the *arsR2M *operon involved in arsenite resistance [21]. 

Mercury resistance in archaea and bacteria is conferred by the *mer *operon involved in detection, regulation, transport, and reduction of Hg(II) [125, 126]. One of the best studied mercury resistance operons in Archaea is the *merRHAI *operon of thermoacidophilic archaeon *Sulfolobus solfataricus *[108, 109]. The operon is under the control of the regulator MerR, which represses the operon in absence of Hg(II) and enhances transcription in its presence. MerH is the metallochaperone with a TRASH (trafficking, resistance, and sensing of heavy metals) domain that binds Hg(II), and MerA is the mercuric reductase for reduction and detoxification to volatile Hg(0) [108, 109]. Some *mer* operons carry additional *mer *genes, notably *merB*, an organomercurial lyase, that cleaves the C-Hg bonds of organomercurials, and the released Hg(II) is reduced to Hg(0) by MerA [126]. Among all haloarchaeal genomes sequenced to date, only *Halobacterium *sp. strain* NRC-1 *and *Haloterrigena turkmenica *have been annotated with *merA* and *merB *genes, respectively [83, 127].

### 3.2. Transporters in Metal Resistance

Membrane transporters may act as the first line of defense against toxic or heavy metals. In order to exert their toxicity, metals need to gain entry within the cell. Thus, the organism may downregulate the transporters responsible for influx or induce the expression of efflux pumps to enable faster removal of toxic metals from within the cell [76]. The use of these membrane transporters and efflux pumps is one of the most common mechanisms of resistance to inorganic ions in microbes.

Both influx and efflux types of transporters for various metals have been annotated in all haloarchaeal genomes sequenced to date (Table 3). The following membrane transporters have been implicated in haloarchaeal metal resistance.

#### 3.2.1. P_1B_-Type ATPases

The P_1B_-type ATPases are a large family of integral membrane proteins driven by ATP hydrolysis [128]. Members of this family are of vital importance to all kingdoms of life, as they generate and maintain electrochemical gradients across membranes by transporting cations and heavy metals [129]. A wide variety of heavy metal ions like Mg(II), Ca(II), Cu(II), Ag(II), Zn(II), and Cd(II) act as substrates to these ATPases [130]. These transporters serve the purpose of uptake (import) of essential elements and efflux (export) of toxic elements, thus conferring resistance to the expelled metal ion [131, 132]. All haloarchaeal genomes have been annotated with metal transporting ATPases. 

A putative Cd(II)-efflux ATPase was annotated on *Halobacterium *sp. strain *NRC-1 *genome [83]. In a system level analysis of *Halobacterium *sp. strain *NRC-1,* the functionality and role of such transporters in metal resistance was shown [22]. They exhibited upregulation of *yvgX*, a *P*_1B_-type ATPase, in response to Cu(II) and Zn(II) metal stress. In bacteria, the *yvgX* family is known to encode two kinds of CopA proteins, CopA1 and CopA2 [133]. CopA1 is essential for copper influx and tolerance, while CopA2 is involved in the influx/efflux of Cu and its transport to Cu-containing enzyme cytochrome oxidase c [133, 134]. A diverse range of organisms contain CopA2-like proteins, suggesting that coding genes appeared early in evolution via gene duplication or horizontal transfer but were kept only in some organisms for a specific biological function [134]. A comparative genome analysis for ATPases in bacteria and archaea showed the preference for CopA2 over CopA1 [10]. It has been proposed that CopA2 may represent the ancestral form of CopA1 protein that may have coevolved with the other metal influx proteins [10]. The *yvgX *of *Halobacterium *sp. strain *NRC-1* was found to be more specific for Cu(II) efflux family as the Δ*yvgX* strain was susceptible to Cu(II) and not to Zn(II) or Co(II) and therefore belongs to the CopA2 family of proteins. CopA2 is also found in other haloarchaea like *Haloarcula marismortui, Haloarcula hispanica, *and *Haloquadratum walsbyi* [135]. 

The As(III)/Sb(III) transporting P_1B_-type ATPase, ArsA discussed in Section 3.1, is present in almost all haloarchaea sequenced to date, including *Halobacterium *sp. strain* NRC-1, Halalkalicoccus jeotgali, Haloarcula hispanica, Natrialba magadii, Haloarcula marismortui, Haloquadratum walsbyi, *and *Natronomonas pharaonis *[135]. ArsB was found to play no role in arsenite resistance in *Halobacterium *sp. strain* NRC-1 *[21]. 

Heavy metal cation-transporting CPx P_1B_-type ATPases are of two types, that is, Cu-CPx-type-ATPases involved in efflux of monovalent cations, Cu(I) and Ag(I), and Zn-CPx-type ATPases involved in the efflux of divalent cations of Zn, Cd and Pb [137–139]. However, Cu-CPx-type ATPases have also been shown to be involved in uptake of copper to meet cellular demands [140, 141]. The *cpx* gene that encodes CPx P_1B_-type ATPases was found to be downregulated by Fe(II), Cu(II), and Ni(II) to avoid influx in *Halobacterium *sp. strain *NRC-1* [22]. This mechanism of resistance involving the downregulation of uptake systems avoids toxic metal buildup within the cell.

#### 3.2.2. Cation Diffusion Facilitators (CDF Family) Metal Transporters

The CDF family of transport proteins is ubiquitously present in all three domains of life [142]. Although CDFs are primarily Zn(II) efflux pumps, bacterial CDFs have been shown to transport Hg(II), Pb(II), Zn(II), Co(II), Fe(II), and Cd(II) from the cytoplasm to the outside of the cell or into subcellular compartments [132, 143]. Based upon their substrate specificity, CDFs have been classified as Zn(II)-CDF, Fe/Zn-CDF, and Mn-CDF [144]. They usually possess six transmembrane domains (TMDs) with a cytoplasmic N- and C-terminal and a histidine loop of variable length between TMD IV and V [145, 146]. The amphipathic domains TMD I, II, V, and VI are involved in metal transfer and are the most conserved, while the hydrophobic TMD III and IV are critical for zinc specificity and mutations within these domains alter substrate specificity [144]. All the proteins of this family of transporters possess a characteristic cation efflux C-terminal domain [147]. These kinds of transporters serve as secondary cation filters in bacteria [132]. The genome of *Halobacterium *sp. strain *NRC-1* was annotated with putative CDF Cd (II) transporter *ZntX*, which confers resistance against Ni(II), Cu(II), and Zn(II) besides Cd(II) [22]. The role of Znt family of CDFs in Cu(II) and/or Zn(II) homeostasis and resistance has been discussed in detail by Haney et al. (2005) [145]. The upregulation of *ZntA *in response to heavy metals (Cu and/or Zn) and poor growth of Δ*zntA* strain in presence of Ni(II), Cu(II), Zn(II), and Cd(II) have confirmed the role of this transporter in metal resistance [22]. The broad specificity of this transporter to various metals has been putatively attributed to the preference of metals by *zntA *based on charge and species rather than size [148]. *Haloarcula hispanica *and* Haloarcula marismortui* have also been annotated with ZntA for Zn(II) transport. A putative CDF family protein has also been found on the chromosome of *Natrialba magadii* for inorganic metal ion transport [135]. 

#### 3.2.3. ATP-Binding Cassette (ABC) Transporters

The multisubunit ABC transporters are one of the largest protein families with a variety of physiological functions. These transporters are ubiquitously present in all living forms from bacteria to eukaryotes including archaea. They are involved in various functions such as nutrient uptake [149], oligopeptide and protein transport [150], metal extrusion [151, 152], and drug efflux [153, 154].

**Figure 3:**
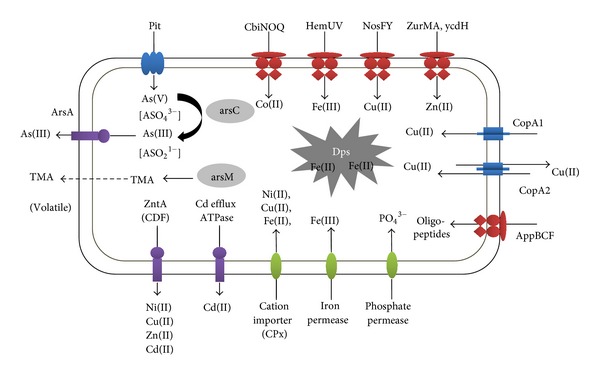
Various transporters playing a role in metal transport, homeostasis maintenance, and resistance in *Halobacterium *sp. strain *NRC-1*, reported to date. The efflux pumps of ATPases and CDF family involved in Cd, Ni, Cu, Zn, Cd, and arsenite transport are represented in purple. ABC transporters (represented in red) involved in metal uptake are many. Certain toxic metals that do not have a dedicated uptake system may gain entry into the cell through other ABC transporters like oligopeptide and phosphate transporters. For example, the arsenate oxyanion gains entry into the cell through the pit/pst phosphate transporters due to its structural similarity to phosphate. The metal ions upon uptake can be detoxified either by enzymatic detoxification (ArsC and ArsM) or by chelation by peptides like Dps (DNA-binding protein of nutrient starved cells).

Although many ABC transporter genes for a variety of substrates have been annotated in all the 10 haloarchaeal genomes sequenced to date, experimentally, very few have been shown to be functional. ABC transporters for sugar and polypeptide have been found in *Haloferax volcanii *[155], *Haloarcula marismortui *[81], *Halobacterium *sp. *NRC-1 *[83], *Natronomonas pharaonis *[156], and *Haloquadratum walsbyi *[80]. All haloarchaea possess at least one copy of metal ion ABC transporter. Some of the ABC transporters in *Halobacterium* sp. *NRC-1* with their functions are listed in Table 4. Many of the ABC transporters are metal ion transporters such as *cbiNOQ* for Co(II) transport [157], *hemUV* for iron uptake [158, 159], *nosFY *for copper [160], and *zurMA *for zinc transport (Figure 3). Although most of the ABC transporter proteins exhibit stringent specificity towards their substrate, a few, such as phosphate transporters, oligopeptide transporters, and dipeptide transporters, have been shown to have multiple specificities and were found to be differentially regulated by more than one metal ion. This has been proposed to facilitate transport of metal ions in addition to their usual function [150]. Kaur et al. (2006) [22] have shown that deletion of transporters like *phoX *(phosphate transport), *appA* (peptide transport), and *ycdH *(Mn(II) transport) along with two putative subunits of Fe(II) transport system does not prove deleterious for *Halobacterium* sp. *NRC-1*. Due to the large repertoire of ABC transport proteins, they concluded that deletion/mutation of a single ABC transporter is easily managed by the organism by substituting the deleted/mutated ABC transporter product with functional ABC transporter product of similar role.

The differential regulation of all three classes of metal transporters discussed above is in congruence with the general norm of micro-organisms utilizing enhanced efflux or decreased influx to resist metals. However, the P_1B_-type ATPases and CDF family have a greater role in maintaining metal homeostasis than the ABC transporters in haloarchaea [22]. 

### 3.3. Transcriptional Changes in Response to Metal Stress

Under unfavourable conditions of growth, all organisms make adjustments at the system level to overcome the stress imposed by the stressor. Presence of heavy metals in their environment triggers global transcriptional regulations either to prevent their entry into the cell or to transform the metal to nontoxic form. This response can be transitory, with perturbations of a few genes within minutes of metal exposure, but once the cell acclimatizes to the new environment, the transcript levels of some early response genes return to preperturbation levels. The early response to a stressor usually results in the upregulation of transcription and translation. As a consequence, the transcripts damaged due to the stressor are replaced and new proteins are synthesized [161, 162].

In haloarchaea, only one study on transcriptional changes in response to heavy metal stress (Fe(II), Cu(II), Co(II), Ni(II), Zn(II), and Mn(II)) in *Halobacterium *sp. strain *NRC-1* has been reported [22]. Besides studying the transcriptional changes by microarray analysis and mutant constructions, Kaur et al. (2006) [22] elucidated a systemic overview to metal stress response, thus providing a snapshot of various mechanisms involved in stress management. A total of 623 genes were found to be differentially regulated in presence of any of the six transition metals used for the study. Around 69% of these genes were early response genes; that is, they exhibited deviation from normal transcript levels within 0–25 minutes of metal exposure. However, 91% of these early response genes transcript levels reverted to preperturbation levels within 25–40 minutes. These included transcriptional regulator genes, transporter genes for phosphate, metals, and peptides, ribosomal protein genes, and protein export genes. Therefore, once the various damaged transcripts and proteins were replaced with the new proteins for managing metal stress and acclimatizing the cells to the new environment, the early response genes were found to revert to preperturbation levels [22].

One of the major toxic effects elicited by heavy metals is the rapid generation of reactive oxygen species (ROS) that damages the cellular machinery [136, 163]. The ROS are usually scavenged by specific enzymatic detoxification systems like superoxide dismutases (SOD), peroxidases, dehydrogenases, and antioxidants like glutathione (GSH) [164]. Therefore, it follows that the genes involved in oxidative stress management are differentially regulated early in the stress management. Model haloarchaeal genomes, including *Halobacterium salinarum* and* Haloferax volcanii*, have been annotated with SOD [165] and catalase-peroxidase (KatG) genes [166]. Metal-induced ROS results in an early increase in the levels of transcripts of genes related to oxidative stress management like dehydrogenases and peroxidases in *Halobacterium *sp. strain *NRC-1* [22].

Few of the early response genes found to be differentially regulated were transcriptional regulators like *tfbB *and *SirR*. TfbB is the transcription initiation factor IIB. Its upregulation indicates a global response towards stress by increasing the rate of transcription to increase protein turnover. Similarly, the upregulation of SirR (silent information regulator) repressed the active uptake of Mn(II), thus providing the organism the ability to overcome the stress. Similar upregulation has also been observed in certain bacteria and yeast [167, 168]. *Staphylococcus aureus *and *Staphylococcus epidermidis *were shown to carry several copies of *sirR* genes that act as divalent metal cation-dependent transcriptional repressor [169]. *cbiN, cbiM,* and *cbiQ *involved in cobalt transport and *zurM, zurA, and ycdH *that encode Mn/Fe ABC-transporters were predicted to be putatively regulated by *sirR* in *Halobacterium *sp. strain* NRC-1* [170]. This was found to be consistent with the observation that *sirR* is essential for survival during metal-induced stress. This was evident from the upregulation of Mn(II) uptake genes *zurM, zurA, and ycdH* in Δ*sirR* strain as compared to parent strain in *Halobacterium *sp. strain *NRC-1*. Thus, in the haloarchaeon *Halobacterium *sp. strain *NRC-1, sirR* acts as a Mn(II)-dependent autorepressor [22]. 

A putative Lrp (leucine-responsive regulatory protein) family protein VNG1197C was reported to upregulate the Cu(II)-P_1B_ type ATPases gene *yvgX *in *Halobacterium *sp. strain *NRC-1* [22]. VNG1197C was found to be a Cu(II)-dependent transcriptional activator carrying a metal-binding TRASH (trafficking, resistance, and sensing of heavy metals) domain. Kaur et al. (2006) [22] proposed that putative metallochaperones VNG0702H and/or VNG2581H deliver Cu(II)/Zn(II) to the TRASH domain of VNG1197C. This binding activates the transcription of *yvgX* as well as the metallochaperones, thus providing Cu(II) resistance to *Halobacterium *sp. strain *NRC-1*. A similar pattern involving a metallochaperone (CopM), a transcriptional regulator with C-terminal TRASH domain (CopT), and a P-type Cu(II) exporting ATPase (CopA) forming the *cop* gene cluster for Cu(II) resistance has been described in *Sulfolobus solfataricus*, a thermoacidophilic crenarchaeon [113, 114].

Thus, organisms have the ability to differentiate between metal ions and therefore elicit responses that enable better survival. These responses could be local or global but in effect would be to efficiently handle the stress. The transcriptional regulation exhibited by *Halobacterium *sp. strain* NRC-1* is an example of how metal homeostasis is maintained. Transient changes in transcripts to resist metals may play a major role in haloarchaea.

## 4. Conclusion

Haloarchaea encounter metals in their natural environment and utilize some of these metals for various key physiological functions. However, at higher concentrations, these metals can be toxic, and thus haloarchaea exhibit metal resistance mechanisms. Knowledge about metal physiology in haloarchaea is cursory, and therefore global studies for gaining insights into the metabolic regulations in response to metal stress are required. Metal physiology studies in model haloarchaeon *Halobacterium *sp. strain* NRC-1* show that they have the ability to elicit a tailor-made response to metal stress. Other genera of the halophilic Euryarchaeota have not yet been subjected to such detailed studies with regard to metal homeostasis and resistance. The development of standard molecular and genetic tools for haloarchaea may facilitate better understanding of the various components involved in metal resistance including detoxifying enzymes, metallochaperones, and metal chelators and transporters. While assessing metal resistance in haloarchaea, the metal speciation should be given importance, as the metal might be unavailable to the cell for uptake, thus giving a higher MIC value. Therefore, beside understanding the molecular mechanisms underlying the resistance to metals, metal speciation and bio-availability studies should be carried out to obtain a complete picture. Further, this could facilitate the use of haloarchaea for bioremediation of metal-polluted hypersaline environments. 

## Figures and Tables

**Table 1:** Various inorganic complexes formed in natural waters, seawater, estuarine waters (variable salinity), and hypersaline waters. As haloarchaea inhabit hypersaline environments where inorganic ligands predominate, inorganic metal speciation is described. The availability of metal depends upon the kind of inorganic complex formed. Lipophilic soluble chlorocomplexes of Hg and Ag are easily available in hypersaline waters. Insoluble (precipitated) ZnCl_2_ and CuCl_2 _are unavailable to the organism. Fe (II), Co (II), Ni (II), and Mn (II) form weak complexes with Cl^−^ that easily dissociate and can be taken up by organisms [28, 41–46].

Metal	Hypersaline (5–35% salinity)	Sea water (3.5% salinity)	Estuarine (variable salinity)	River water/natural water
Cd	CdCl_2_, CdCl^+^	CdCl^+^	CdCl_2_, CdCl^+^	Cd^2+^, CdCO_3_
Ag	AgCl^0^, AgCl^2−^, AgCl_3_^2−^, AgCl_4_^3−^	AgCl^0^, AgHS^0^	AgCl^0^, AgHS^0^, AgCl^2−^, AgCl_3_^2−^, AgCl_4_^3−^	Ag^+^, AgCl^0^
Hg	HgCl^0^, HgCl^−^, HgCl_4_^2−^	HgCl^−^	HgCl^0^, HgCl^−^, HgCl_4_^2−^	Mixture of Hg- chloro and hydroxy complex
Zn	ZnCl_2_	Zn^2+^, ZnCl_2_, ZnCO_3_, Zn(HCO_3_)_2_, Zn(OH)_2_, ZnSO_4_	Zn^2+^, ZnCl_2_, ZnCO_3_, Zn(HCO_3_)_2_, Zn(OH)_2_, ZnSO_4_	Hydrated Zn^2+^
Cu	CuCl_2_	Carbonato and hydroxy complexes	CuCl_2_, Carbonato and hydroxy complexes	Cu^2+^, CuCO_3_

**Table 2:** Bioavailability of metal-ligand complexes in hypersaline conditions depending upon the nature of the complex formed.

Availability	Type of complex
Biologically unavailable	(i) Strong insoluble inorganic metal-chloro complexes (ZnCl_2_, CuCl_2_)
	(ii) Soluble not easily dissociable metal-chloro complexes (CdCl_2_)
	(iii) Biosorbed metal complexes (i.e., metals sorbed on a biotic ligand)

Biologically available	(i) Strong soluble lipophilic inorganic metal-chloro complexes (AgCl^2−^, AgCl_3_^2−^, AgCl_4_^3−^, and HgCl_2_)
	(ii) Weak metal-chloro complexes (Fe, Co, Ni, and Mn)
	(iii) Metal complexes sorbed to abiotic ligands

**Table 3:** Annotated transporters for various metals in haloarchaeal genomes. Ten haloarchaeal genomes have been completely sequenced while others are partially sequenced. All these organisms have been annotated with transporters belonging to the following type of transporters-cation efflux type, P_1B_-type ATPases, cation diffusion facilitator (CDF) family, and ATP-binding cassette (ABC) family. The most abundant transporters were for iron followed by copper. Only one haloarchaeon, *Halogeometricum borinquense*, was annotated with silver transporters [135].

Transporters for metals	*H.s. *	*H.m. *	*H.v. *	*H.w. *	*H.l. *	*H.mu. *	*H.u. *	*H.b. *	*H.t. *	*H.j. *	*N.p. *	*N.m. *
Copper	+	+	+	+	+	+	+	+	·	+	+	+
Iron	+	+	+	+	+	+	+	+	+	+	+	+
Manganese	·	+	+	+	+	·	·	·	+	·	+	+
Zinc	+	+	+	+	+	+	+	·	+	+	+	+
Cobalt	+	+	+	+	+	+	+	+	+	+	+	+
Nickel	+	·	·	+	·	·	·	+	·	+	+	+
Molybdenum	·	+	+	·	·	·	·	·	·	·	·	·
Arsenic	+	+	+	+	+	+	+	·	+	+	·	·
Cadmium	+	+	·	+	·	·	·	·	·	·	+	+
Magnesium	·	+	+	·	·	·	+	+	·	+	·	·
Silver	·	·	·	·	·	·	·	+	·	·	·	·

(+) present; (·) not annotated yet; *H.s., Halobacterium *sp. strain *NRC-1; H.m., Haloarcula marismortui; H.v., Haloferax volcanii; H.w., Haloquadratum walsbyi; H.l., Halorubrum lacusprofundi; H.mu., Halomicrobium mukohataei; H.u., Halorhabdus utahensis; H.b., Halogeometricum borinquense; H.t., Haloterrigena turkmenica; H.j.,  Halalkalicoccus jeotgali; N.p., Natronomonas pharaonis; N.m., Natrialba magadii.*

**Table 4:** ABC transporters with various functions present in some model haloarchaea. ABC transporters have three components that together help in uptake of nutrients or for the efflux of extracellular proteins, enzymes, and toxicants. Permease is the transmembrane component and is responsible for the uptake of ions or macromolecules, while the ATP-binding component is the water soluble domain that binds ATP. Substrate binding at the substrate binding site brings about a conformational change in the ATP-binding component resulting in ATP hydrolysis. The presence or absence of the three components of ABC transporters for sugar, peptide, amino acids, phosphate, and iron transport is shown in the following table [135, 136].

ABC transporters	*H.s. *	*H.v. *	*H.m. *	*H.w. *	*H.l. *	*N.p. *	*N.m. *
Sugar transport system components							
Permease	+	+	+	+	−	−	−
ATP binding	+	+	+	+	−	−	−
Substrate binding	−	−	+	+	+	−	−
Phosphate transport system components							
Permease	+	+	+	+	+	+	+
ATP binding	−	+	+	+	+	+	+
Susbtrate binding	+	+	+	+	+	+	+
Dipeptide/oligopeptide transport system components							
Permease	+	+	+	+	−	+	−
ATP binding	+	+	+	+	+	+	+
Susbtrate binding	−	−	+	+	−	+	+
Amino acid transport system components							
Permease	−	+	+	+	−	+	−
ATP binding	+	+	+	+	−	+	−
Susbtrate binding	−	+	−	+	+	+	+
Fe(III) transport system components							
Permease	+	+	+	+	−	+	+
ATP binding	+	+	−	−	−	+	−
Susbtrate binding	−	−	−	+	+	+	−

*H.s., Halobacterium *sp. strain* NRC-1; H.v., Haloferax volcanii; H.m., Haloarcula marismortui; H.w. Haloquadratum walsbyi; H.l., Halorubrum lacusprofundi; N.p, Natronomonas pharaonis; Natrialba magadii;* (+) present; (−) absent.
